# Vacuoles, E1 Enzyme, X-linked, Autoinflammatory, and Somatic (VEXAS) Syndrome: A Case Report

**DOI:** 10.7759/cureus.78373

**Published:** 2025-02-02

**Authors:** Maheen Zaidi, Foster West, Zachary Ellis, Ryan A Yates, Harrison Davis

**Affiliations:** 1 Internal Medicine, Baptist Memorial Hospital, Oxford, USA; 2 Hematology and Oncology, Baptist Memorial Hospital, Oxford, USA

**Keywords:** doxycycline, fever of unknown origin (fuo), myelodysplastic syndromes, uba1 mutation, vexas

## Abstract

VEXAS is an acronym that stands for the technical terms of key descriptors of the condition: vacuoles, E1 enzyme, X-linked, autoinflammatory, and somatic (VEXAS) syndrome, which is a recently identified autoinflammatory disorder primarily affecting men older than 50 years of age. It is commonly associated with a somatic mutation in the X-linked ubiquitin-activating enzyme-encoding *UBA1* gene. This condition manifests in a range of hematologic and systemic inflammatory symptoms, such as cytopenias, recurrent fevers, and an elevated risk for hematologic malignancies like myelodysplastic syndrome (MDS). Currently, glucocorticoids are the mainstay of treatment; however, biological therapies like janus kinase (JAK) and interleukin (IL) inhibitors have had varying degrees of effectiveness; unfortunately, despite these treatments, symptoms frequently persist and reoccur. Allogenic bone marrow transplantation is currently being considered as a possible treatment.

This is a case of a 76-year-old male who presented with an unusual presentation of VEXAS syndrome and one who temporarily responded to treatment with doxycycline, an antibiotic with well-known anti-inflammatory qualities. This case report highlights our patient’s unique presentation of VEXAS syndrome and emphasizes the importance of multiple bone marrow samples when the diagnosis is unclear. In this case, the results of the initial biopsy were inconclusive; however, following repeat bone marrow biopsy and the diagnosis of MDS, VEXAS syndrome was confirmed with testing for *UBA1* mutations. This case underscores the importance of early and routine *UBA1* mutation testing to facilitate timely diagnosis, improve patient outcomes, and guide the development of more effective treatment strategies. It also contributes to a broader understanding of VEXAS syndrome and its potential therapeutic approaches within the medical community.

## Introduction

Vacuoles, E1 enzyme, X-linked, autoinflammatory, and somatic (VEXAS) syndrome is a rare, recently discovered autoinflammatory condition. This condition presents predominately in men in the fifth decade of life or later. VEXAS is an acronym describing the characteristic traits of the syndrome: V for vacuoles, which are found primarily in myeloid and erythroid precursor cells; E and X represent the E1 enzyme encoded by the X-linked ubiquitin-activating enzyme 1 (*UBA1*) gene; and A and S represent the autoinflammatory and somatic nature of the syndrome [1]. Through a unique genome-driven approach, VEXAS syndrome was first discovered in 2020 through exome sequencing of over 141,000 men and women. The genome sequencing identified 25 men with a mutation in the X-linked *UBA1* gene [1].

The men identified with this mutation had similar inflammatory and hematologic symptoms, including recurrent fevers and cytopenia. This newly identified syndrome unified the vague and seemingly random findings these men had been experiencing without a previous diagnosis or explanation. While most systemic autoinflammatory disorders originate from inherited mutations, the mutation in VEXAS syndrome appears to be acquired later in life [2]. The presentation of VEXAS syndrome varies significantly among affected individuals. Various organ systems, including skin, lungs, blood vessels, bone marrow, and cartilage, can be affected. This results in some of the most common presentations: fever, polychondritis, giant cell arteritis, macrocytic anemia, thrombocytopenia, and progressive bone marrow failure. Secondary to the myeloid origin of the mutation, there is an increased risk for hematologic malignancy such as myelodysplastic syndrome (MDS) and multiple myeloma (MM). Current literature suggests a 25% to 55% increase in the risk of MDS in patients with VEXAS syndrome [1]. There is currently no standardized treatment or cure for VEXAS syndrome [3,4]. Glucocorticoids have historically been the primary treatment for managing symptoms. However, patient symptoms are frequently refractory to treatment with steroids [1]. Therefore, various biological therapies, including janus kinase (JAK) and interleukin (IL) inhibitors, have been trialed with varying success. Allogenic bone marrow transplant is optimistically proposed as a possible curative solution in the future [1,3,4]. Several recent publications on VEXAS syndrome have encouraged the medical community to combine efforts and establish a more standardized and effective strategy for managing symptoms and treating the underlying syndrome [4].

## Case presentation

A 76-year-old Caucasian male presented to the clinic for evaluation of low-grade fevers, generalized malaise, and unintentional weight loss of four weeks duration. The initial workup was unremarkable, except for an elevated erythrocyte sedimentation rate (ESR). He was instructed to continue supportive care at home and follow-up outpatient. Unfortunately, he was hospitalized one month later due to persistent systemic symptoms. An extensive workup was initiated to determine the etiology behind his recurrent fevers. Labs on admission were remarkable for pancytopenia and elevated inflammatory markers, an elevated lactate dehydrogenase (LDH), ESR, C-reactive protein (CRP), and ferritin (Table 1). No blasts were noted on the differential.

**Table 1:** Significant labs during initial outpatient presentation and first hospital admission. WBC: white blood cells, Hgb: hemoglobin, Plts: platelets, ESR: erythrocyte sedimentation rate, CRP: c-reactive protein, and LDH: lactate dehydrogenase.

Lab	Initial encounter	Hospitalization 1 month later	Reference range
WBC	5.4	4.8	5-10 K/uL
Hgb	10.6	8.5	12-16 g/dL
Plts	142	101	150-500 K/uL
Blasts	None	None	<5%
ESR	128	130	<20 mm/h
CRP	-	14.7	<5 mg/L
LDH	-	221	100-190 IU/L
Ferritin	-	3538	25-335 ng/mL
Copper	-	193	70-140 mcg/dL
Zinc	-	44	60-120 mcg/dL

**Figure 1:**
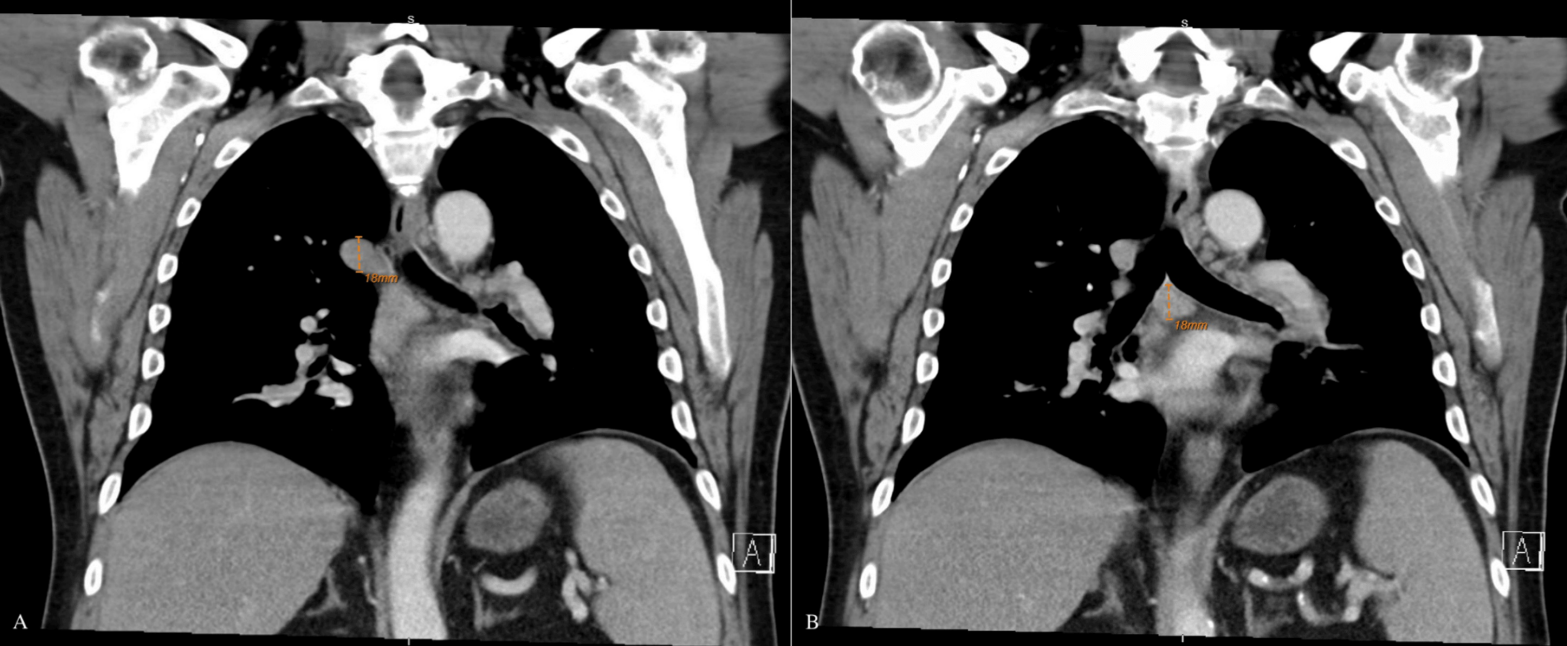
CT chest. A) CT chest showing hilar lymph node B) CT chest showing pre-carinal lymph node. CT: Computed tomography.

Computed tomography (CT) of the chest showed multiple prominent mediastinal lymph nodes and a 1.8 cm left hilar lymph node (Figure 1). A bronchoscopy was performed to obtain biopsies of the hilar lymph. The bronchoscopy showed irregularity and fullness in the left upper lobe mucosa and carina. However, pathology, cytology, and cytometry were unremarkable and negative for any malignant cells. The patient was treated supportively during admission but showed minimal symptomatic improvement on non-steroidal anti-inflammatory drugs (NSAIDs) and was therefore discharged on prednisone 20 mg daily for three days with close outpatient follow-up. 

At outpatient follow-up shortly after discharge, the patient continued to report intermittent fevers (reported home temperature readings of 99-103º F), which occurred after completing the course of glucocorticoids. Doxycycline (100 mg twice daily) was initiated empirically for 14 days due to concern for possible infectious etiology of persistent fevers. Serologies for lyme disease, ehrlichiosis, and other tick-borne illnesses were negative. A referral to hematology-oncology was placed for evaluation and consideration of bone marrow biopsy as there was a concern for an underlying hematological condition. However, the patient reported significant improvement in symptoms after taking doxycycline, including resolution of fever.

Unfortunately, several weeks after feeling back to baseline, he was hospitalized again for recurrent symptoms. The recurrence and severity of his symptoms led to consultations with multiple subspecialties and prompted further infectious and hematologic evaluations. Our infectious evaluation yielded unremarkable results, with negative tests for ehrlichia, anaplasma, babesia, lyme, coxiella, bartonella, brucellosis, mycoplasma, tuberculosis, endemic mycoses, human immunodeficiency virus (HIV), hepatitis B/C, syphilis via rapid plasma reagin (RPR), Epstein-barr virus (EBV) PCR, and cytomegalovirus (CMV) PCR. The only positive finding was rickettsia IgG. Our rheumatologic evaluation was remarkable for an elevated ESR, CRP, and rheumatoid factor (RF). Otherwise, the antinuclear antibody (ANA) and antineutrophil cytoplasmic antibody (ANCA), as well as complements and uric acid, were negative. From a hematologic oncologic perspective, previous fine needle aspiration (FNA) of the hilar lymph node and flow cytometry from previous admission revealed no pathology. However, the positron emission tomography (PET) scan showed moderate uptake in mediastinal and hilar lymph nodes, increased moderate splenic uptake, a 1.3 cm right thyroid nodule, and a few nodular foci of mild-moderate uptake in the pancreas. Since his fevers previously improved significantly on doxycycline, suspicion of an unidentified infectious cause was presumed, and doxycycline was restarted during hospitalization. After initiation of doxycycline, he remained afebrile and was, therefore, discharged with outpatient follow-up. However, once he completed the course of doxycycline, his fevers recurred. He underwent a bone marrow biopsy as an outpatient three months after the initial presentation. Bone marrow biopsy revealed hypercellular marrow, increased myeloid elements, and decreased erythroid precursors and next-generation sequencing (NGS) with *ZRSR2 *mutation. He was also later found to have left lower extremity and right upper extremity deep vein thrombosis (DVT) and was started on xarelto. Repeated courses of doxycycline and other antibiotics did not fully alleviate his fevers and systemic symptoms.

Over the four to five months after the initial presentation, the patient was hospitalized multiple times for severe symptomatic normocytic anemia without any apparent bleeding. He required multiple packed red blood cell (RBC) transfusions for severe acute anemia. At this time, a repeat bone marrow biopsy was performed due to high suspicion of myelodysplastic syndrome, which showed low blasts, vacuolated myeloid, and erythroid precursors consistent with MDS.

**Figure 2:**
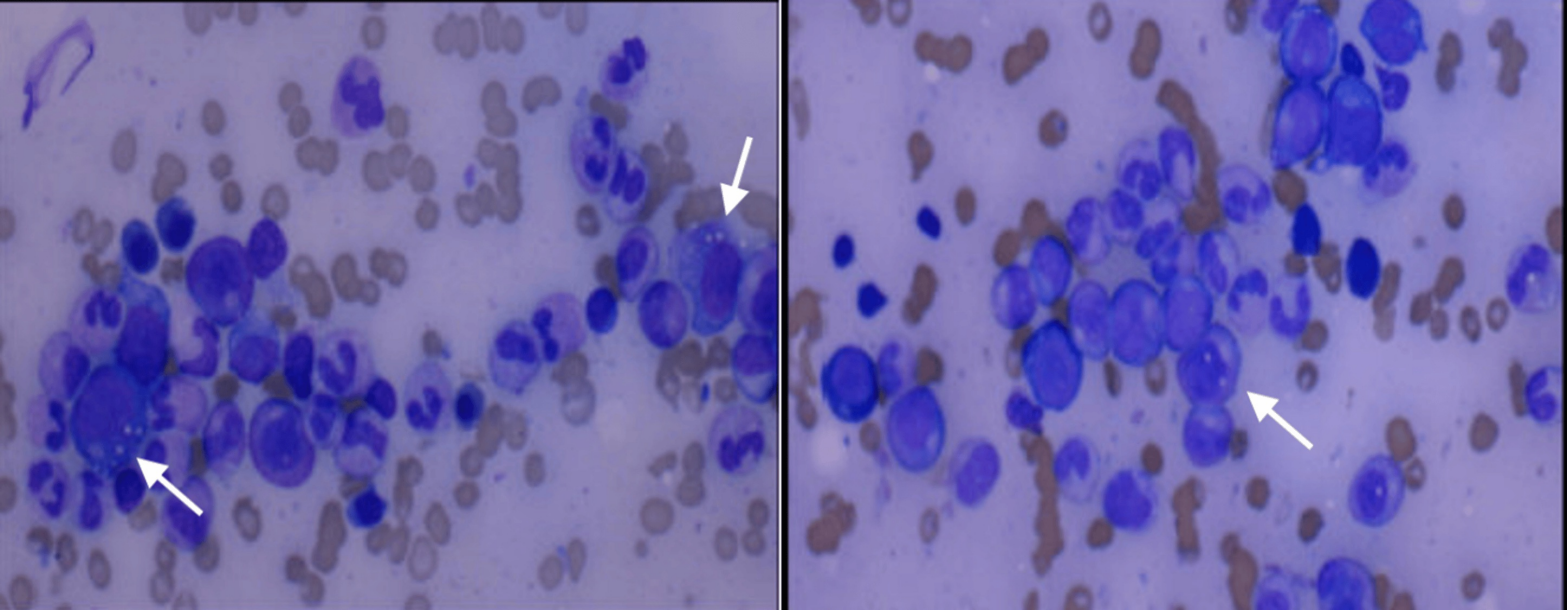
Bone marrow biopsy showing vacuolization (arrows) of myeloid and erythroid precursor cells.

Due to the presence of vacuolization, testing for the ubiquitin-like modifier activating enzyme 1 (*UBA1*) gene was conducted, yielding a positive result and confirming the diagnosis of VEXAS syndrome (Figure 2). Since anemia was the primary manifestation at that time, erythropoietin was initiated. Doxycycline was discontinued, and prednisone 40 mg was started, which led to the complete resolution of his fevers and systemic symptoms. He was referred to a tertiary care center for evaluation of allogeneic stem cell transplantation and further management of his VEXAS Syndrome.

Since the diagnosis, the patient’s hemoglobin has remained stable on erythropoietin infusions, and he has not required any further transfusions or had any further hospitalization for acute anemia. His prednisone was tapered to 20 mg with control of symptoms. In February 2024, he was started on fludrabine/busulfan/cyclophosphamide (Flu/Bu/Cy) conditioning for a mismatch unrelated donor (MMUD) stem cell transplant.

A day after beginning Flu/Bu/Cy conditioning, while steroids were being weaned, he had a recurrence of fever, nausea, vomiting, and cough. He was noted to have a flare of his VEXAS syndrome, and therefore, the transplant was aborted due to the inability to wean from the steroids. To date, the patient remains glucocorticoid-dependent, requiring moderate doses of prednisone. To facilitate steroid weaning and enable conditioning for transplant, ruxolitinib 5 mg twice daily was recently introduced to decrease his reliance on glucocorticoids.

## Discussion

VEXAS syndrome is a rare autoinflammatory syndrome recently discovered through exome-driven sequencing [2]. There may be a higher prevalence than initially suspected, so documentation of each case is vital to build the medical community’s understanding. Some aspects of the case mentioned above represent the typical presentation of VEXAS syndrome, while other findings were more unique and worthy of specific mention. This patient fits the typical demographic as he was a man who began experiencing symptoms after his fifth decade of life [1]. The patient’s recurrent fevers, symptomatic anemia, leukopenia, increased inflammatory markers, and eventual diagnosis of MDS are all typical presentations of VEXAS syndrome [1,2]. MDS was present in 25%-55% of the cases [1]. In this case, while the initial bone marrow biopsy was unremarkable, the second bone marrow biopsy showed vacuolated myeloid and erythroid precursors, which diagnosed MDS. The presence of vacuoles on biopsy raised suspicion for VEXAS syndrome, leading to testing for the *UBA1* mutation. This resulted in a prolonged workup by multiple specialists, including hematology, pulmonology, infectious disease, and rheumatology. Ongoing publication of literature detailing the diverse symptoms in these patients may contribute to earlier recognition and diagnosis.

Glucocorticoids are well-documented as the current mainstay of treatment. Although helpful, symptoms often recur on glucocorticoid treatment. Current literature expresses the need for better treatment regimens. Allogenic bone marrow transplant appears to be the most promising possible curative treatment [1,4]. The patient is currently receiving glucocorticoids and awaiting a potential stem cell transplant. A distinctive aspect of this case is the patient’s response to doxycycline. Initially, doxycycline led to a complete resolution of symptoms, which would recur upon discontinuation. Subsequent courses provided diminishing relief, and over time, doxycycline became ineffective. We believe this may have been secondary to the known anti-inflammatory properties of doxycycline [5]. From our literature review, it does not appear that antibiotics have been previously documented to alleviate symptoms of VEXAS syndrome. This patient’s response to doxycycline may help us better understand possible etiology and management. This case suggests infection could have played a role in initially precipitating the disease. Although antibiotic therapy is unlikely to become the much-needed mainstay of treatment, this patient’s response was unique, which might help with the overall understanding of how to manage this complex syndrome.

A distinctive aspect of this case was the decision to perform a second bone marrow biopsy. While the initial biopsy yielded no significant findings, the clinician's suspicion of an underlying bone marrow disorder, prompted by the recurrence of symptoms, led to the second biopsy. The presentation of bone marrow biopsies can vary, with some showing only minimal changes [4]. The second biopsy ultimately confirmed the diagnosis of MDS, which then led to *UBA1* mutation testing and, finally, the diagnosis of VEXAS syndrome. Documenting the need for a second biopsy is crucial, as it may assist future clinicians facing similar cases where the initial bone marrow biopsy is non-diagnostic, but symptoms persist.

Finally, this brings us to the exome-driven approach initially taken in discovering VEXAS syndrome [2]. The disease presents with many systemic, inflammatory, and seemingly unrelated symptoms. Although the pathology observed in the bone marrow biopsy appears somewhat more consistent than the range of systemic symptoms, our case provides additional evidence that bone marrow biopsy results can vary. However, the presence of the *UBA1* mutation is a hallmark of VEXAS syndrome in all patients [1]. This case should serve as a reminder for clinicians to consider *UBA1* gene testing earlier and more regularly. This would lead to more documented cases, allowing the medical community to understand the syndrome better and continue efforts for better treatment options. Earlier diagnosis would avoid confusion and frustration for both the patient and the clinician.

## Conclusions

This is an interesting and unique case of a recently discovered disease called VEXAS syndrome. This specific patient was unique in that his symptoms would initially fully resolve with treatment with doxycycline for weeks at a time. However, each course of treatment with doxycycline yielded a less complete resolution of his symptoms. This is likely partly due to the anti-inflammatory properties of doxycycline, though it may also suggest a potential infectious trigger for the disease. The patient initially had an unremarkable bone marrow biopsy, followed by a repeat biopsy that revealed classic vacuolization of myeloid and erythroid precursor cells. This underscores the importance of ongoing evaluation when a diagnosis is not immediately evident.
